# Circulating free DNA concentration as a marker of disease recurrence and metastatic potential in lung cancer

**DOI:** 10.1186/s40169-019-0229-6

**Published:** 2019-04-18

**Authors:** Hanifeh Mirtavoos-Mahyari, Soudeh Ghafouri-Fard, Adnan Khosravi, Elahe Motevaseli, Zahra Esfahani-Monfared, Sharareh Seifi, Babak Salimi, Vahid Kholghi Oskooei, Mohsen Ghadami, Mohammad Hossein Modarressi

**Affiliations:** 1 Department of Medical Genetics, Faculty of Medicine, Tehran University of Medical Sciences, Tehran, Iran; 2 Department of Medical Genetics, Shahid Beheshti University of Medical Sciences, Tehran, Iran; 3 Tobacco Prevention and Control Research Center, National Research Institute of Tuberculosis and Lung Diseases (NRITLD), Shahid Beheshti University of Medical Sciences, Tehran, Iran; 4 Department of Molecular Medicine, School of Advanced Technologies in Medicine, Tehran University of Medical Sciences, Tehran, Iran; 5 Chronic Respiratory Diseases Research Center, National Research Institute of Tuberculosis and Lung Diseases (NRITLD), Shahid Beheshti University of Medical Sciences, Tehran, Iran

**Keywords:** Circulating free DNA, Lung cancer, Metastasis

## Abstract

**Background:**

Plasma circulating cell-free (cf) DNA is regarded as a source of tumor DNA. Based on availability of blood tissue for the purposes of early detection of cancer and patients’ follow-up, several studies have evaluated concentration of cf DNA in cancer patients in association with tumor features. In the present study, we assessed concentration of cf DNA in lung cancer patients with two commercial kits (MN and QIAGEN) to find whether it can be used as a prognostic biomarker.

**Results:**

Primary cf DNA concentrations as measured by QIAGEN kit was significantly higher in patients who died in the follow-up period compared with alive patients (P = 0.007). Moreover, the concentrations as measured by both methods were higher in patients who experienced recurrence in the follow-up period compared with patients without recurrence (P = 0.008 and 0.007 for MN and QIAGEN kits respectively). Significant associations were also found between cf DNA concentrations and tumor stage (P = 0.005 and 0.02 for MN and QIAGEN kits respectively). Notably, cf DNA concentration was higher in metastatic tumors compared with non-metastatic tumors in association with number of involved organs. Based on the AUC values, both kits could differentiate metastatic cancers from non-metastatic ones with accuracy of 98%.

**Conclusions:**

The current study highlights the accuracy of cf DNA concentrations for prediction of disease course in lung cancer patients.

## Introduction

Circulating cell-free (cf) DNA is thought to be secreted in the blood circulation through necrosis, apoptosis or active release from nucleated cells. Inflammation, trauma and malignancy have been linked with elevated concentrations of cfDNA [1]. Based on the observed release of DNA from tumoral cells during processes such as necrosis and apoptosis, plasma cfDNA is regarded as a source of tumor DNA for the purposes of early detection of cancer and patients’ follow-up [2]. High mortality rate of lung cancer and poor patients’ outcome have prompted researchers to find suitable biomarkers for this kind of cancer. High cf DNA concentrations at baseline have been associated with worse patients’ outcome of cancer patients in some studies [2]. Moreover, the clinical validity of measurement of cfDNA for the estimation of lung cancer survival has been confirmed through meta-analysis of available literature [3]. Besides, quantification of cfDNA has a diagnostic accuracy comparable with conventional blood-based biomarkers for lung cancer screening [4]. Moreover, specific mutations found in cfDNA could act as prognostic and predictive biomarkers for patients with non-small cell lung cancer (NSCLC). For instance, the presence of EGFR activating mutations in cfDNA of these patients has been regarded as a predictive marker for response to EGFR-tyrosine kinase inhibitors [5]. Specifically, the L858R EGFR mutation in cfDNA could predict the overall survival of NSCLC patients [6].

Although several studies have quantified circulating cfDNA in lung cancer patients, no definite evidence indicates the associations between its concentrations and tumor features. The inconsistencies between the results of former studies might be originated from the method of cfDNA quantification. Consequently, in the present study we aimed at quantification of cfDNA in Iranian lung cancer patients with two commercially available kits to find the associations between its concentration and patients’ clinical data in a comparative manner.

## Materials and methods

### Patients

A total of 44 patients with NSCLC participated in the current study. Patients were hospitalized in Masih Daneshvari Hospital, Tehran during April 2017 to May 2018. The recurrence after initial treatment and patients’ outcome were recorded. All patients had performance status of 1–2 based on the Eastern Cooperative Oncology Group (ECOG) criteria [7]. The study protocol was approved by ethical committee of Tehran University of Medical Sciences. Written informed consent forms were signed by all study participants.

### Assessment of free DNA concentrations

Venous blood was gathered in sterile EDTA-coated tubes. Samples were centrifuged at 590×*g* for 15 min in room temperature. Isolated plasma samples were kept at − 80 °C. cfDNA was extracted from plasma samples using the QIAamp Circulating Nucleic Acid Kit (QIAGEN, Valencia, CA, USA) and NucleoSpin Plasma XS (MN, Germany). cfDNA concentrations were quantified using NanoDrop 2000 (Thermo Scientific). Total concentrations of cf DNA in the plasma were reported. The same volume of plasma was used for cf DNA extraction from all samples (2 ml for extraction with QIAGEN kit and 260 µl for extraction with MN kit based on the guidelines provided by the companies).

### Statistical analyses

Statistical analyses were performed in SPSS v.18.0 (IBM Corp., Armonk, NY, USA). Data were reported as mean ± standard deviation (SD). Paired-samples t test was used for comparison of concentrations of cfDNA in each sample as measured by each kit. The significance of association between cfDNA concentrations and tumor features was assessed using independent-samples t test. For all statistical analyses, P < 0.05 was considered as significant. The receiver operating characteristic (ROC) curve was designed to evaluate the suitability of cfDNA concentrations for prediction of recurrence probability and metastatic potential. The Youden index (j) was used to maximize the difference between sensitivity (true-positive rate) and 1—specificity (false-positive rate).

## Results

### General clinical information of patients

The current study included 44 patients with NSCLC. Patients were followed up until October 2018. During follow-up period 12 of them died. Blood samples were taken from patients before any cancer treatment at initial visit. Table 1 shows General demographic and clinical data of patients.

**Table 1:** General demographic and clinical data of patients

Variables	Values
Age (mean ± SD (range))	59.79 ± 11.65 (34–84)
Cell free DNA concentration—MN Kit (mean ± SD (range)) (ng/ml)	13.92 ± 4.11 (3.6–22.3)
Cell free tumor DNA—QIAGEN Kit (mean ± SD (range)) (ng/ml)	19.1 ± 6.03 (6.1–32.6)
Recurrence after (month) (mean ± SD (range))	7.76 ± 5.4 (1–19)
Gender	
Male	45.5%
Female	54.5%
Smoking	
Yes	22.7%
No	77.3%
Stage	
II	2.3%
III	4.5%
IV	93.2%
Recurrence	
Yes	56.8%
No	43.2%
Current status	
Alive	67.6%
Dead	32.4%
Metastasis	
Non metastatic	11.4%
Metastasis to one organ	68.1%
Metastasis to two organ	9.1%
Metastasis to three organ	11.4%

### Comparison of circulating free DNA concentrations as detected by two methods

The mean values (± standard deviation) of circulating DNA concentrations as measured simultaneously by two methods were 13.72 (3.95) (ng/ml) and 19.1 (6.03) (ng/ml) for MN and QIAGEN kits respectively (P < 0.001).

### Association between circulating free DNA concentration and patients’ characteristics

**Fig. 1:**
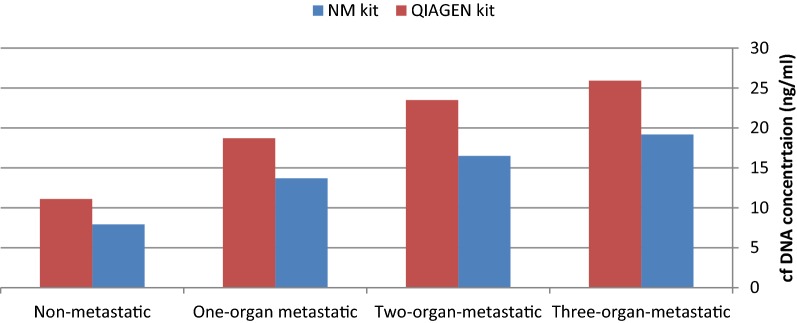
Circulating free DNA concentrations (ng/ml) in association with metastasis potential

Primary cfDNA concentrations (ng/ml) as measured by QIAGEN kit was significantly higher in patients who died in the follow-up period compared with alive patients (P = 0.007). Moreover, the concentrations as measured by both methods were higher in patients who experienced recurrence in the follow-up period compared with patients without recurrence (P = 0.008 and 0.007 for MN and QIAGEN kits respectively). Significant associations were also found between cfDNA concentrations and tumor stage (P = 0.005 and 0.02 for MN and QIAGEN kits respectively). Notably, cfDNA concentration was higher in metastatic tumors compared with non-metastatic tumors in association with number of involved organs (Fig. 1 and Table 2). Table 2 shows associations between cfDNA concentration and patients’ characteristics.

**Table 2:** Association between cf DNA concentration (ng/mL) and patients’ characteristics

	cfDNA concentration (MN kit) (ng/ml)	P value	cfDNA concentration (QIAGEN kit) (ng/ml)	P value
Gender				
Male vs. female	13.96 (4.67) vs. 13.88 (3.69)	0.94	19.21 (6.59) vs. 19.01 (5.67)	0.91
Smoking				
Yes vs. no	13.96 (4.55) vs. 13.9 (4.05)	0.97	19.17 (6.1) vs. 19.08 (6.1)	0.96
Current status				
Alive vs. dead	13.22 (4.56) vs. 16.21 (3.4)	0.05	17.56 (5.78) vs. 23.49 (6.28)	0.007
Recurrence				
Yes vs. no	15.81 (3.45) vs. 12.06 (4.7)	0.008	21.93 (6.09) vs. 16.27 (5.7)	0.007
Stage				
II and III vs. IV	7.66 (2.5) vs. 14.37 (3.84)	0.005	11.43 (2.05) vs. 19.66 (5.84)	0.02
Metastasis				
Non metastatic vs. Metastasis to one organ	7.94 (3.81) vs. 13.69 [3]	0.02	11.1 (3.52) vs. 18.71 (4.73)	0.01
Non metastatic vs. Metastasis to two organs	7.94 (3.81) vs. 16.5 (2.29)	0.001	11.1 (3.52) vs. 23.5 (4.98)	0.002
Non metastatic vs. Metastasis to three organ	7.94 (3.81) vs. 19.18 (3.45)	< 0.001	11.1 (3.52) vs. 25.92 (6.24)	< 0.001
Metastasis to one organ vs. Metastasis to two organs	13.69 [3] vs. 16.5 (2.29)	0.33	18.71 (4.73) vs. 23.5 (4.98)	0.26
Metastasis to one organ vs. Metastasis to three organs	13.69 [3] vs. 19.18 (3.45)	0.004	18.71 (4.73) vs. 25.92 (6.24)	0.01
Metastasis to two organs vs. Metastasis to three organs	16.5 (2.29) vs. 19.18 (3.45)	0.57	23.5 (4.98) vs. 25.92 (6.24)	0.87

After partial correction for patients’ gender, circulating DNA concentrations (ng/ml) were not correlated with either age of patients or recurrence time after initial diagnosis (Table 3).

**Table 3:** Partial correlation between free DNA concentrations (ng/ml) and age/recurrence time (controlled for gender)

	cfDNA concentration (MN kit) (ng/ml)		cfDNA concentration (QIAGEN kit) (ng/ml)	
	R	P value	R	P value
Age	− 0.23	0.06	− 0.18	0.12
Recurrence time	− 0.05	0.4	− 0.19	0.2

### ROC curve analysis

**Fig. 2:**
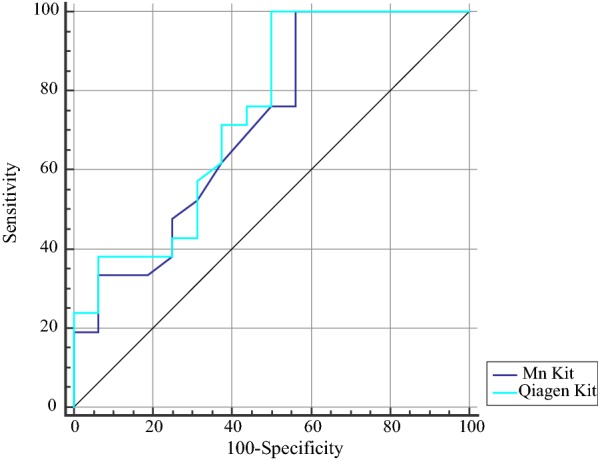
ROC curve analysis for assessment of diagnostic power of cf DNA concentrations for differentiation of recurrence probability

**Fig. 3:**
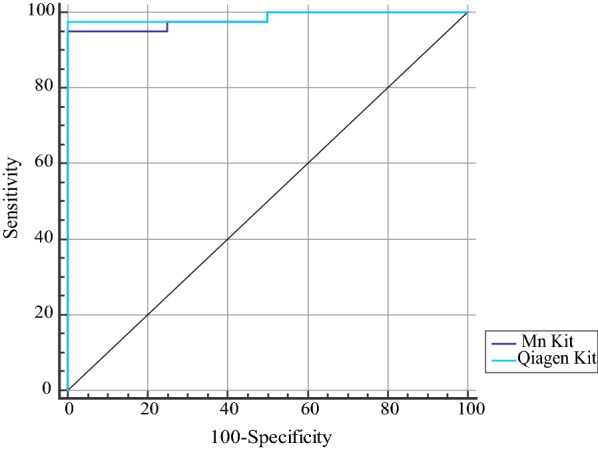
ROC curve analysis for assessment of diagnostic power of cf DNA concentrations (ng/ml) for differentiation of metastatic potential

We evaluated diagnostic power of cfDNA concentration for prediction of recurrence probability and metastatic potential (Table 4). Both kits had 100% sensitivity for differentiation of recurrence probability (Fig. 2). Based on the AUC values, both kits could differentiate metastatic cancers from non-metastatic ones with accuracy of 98% (Fig. 3).

**Table 4:** The results of ROC curve analysis

	Differentiation of recurrence probability						Differentiation of metastatic potential					
	Estimate criterion	AUC	J^a^	Sensitivity	Specificity	P-value^b^	Estimate criterion	AUC	J^a^	Sensitivity	Specificity	P-value^b^
MN	> 11.3	0.71	0.43	100	43.7	0.01	> 10.1	0.98	0.95	95	100	< 0.001
QIAGEN	> 15.1	0.74	0.5	100	50	0.004	> 13	0.98	0.97	97.5	100	< 0.001

^a^Youden index, ^b^Significance level P (Area = 0.5), Estimate criterion: optimal cut-off point for cf DNA concentration

## Discussion

Lung cancer is a human malignancy associated with high mortality rate. Several prognostic factors including tumor stage, performance status, age at diagnosis and response to first-line chemotherapy have been recognized for this kind of cancer [8]. In the present study, we first quantified cfDNA levels in lung cancer patients and then assessed associations between cfDNA concentrations and some of known prognostic factors for lung cancer. Previous studies have measured cfDNA levels using different methods such as spectrophotometry [9], digital polymerase chain reaction (PCR)-based technologies [10] or Alu81-quantitative PCR [11]. We used two commercially available kits for isolation of cfDNA and subsequently quantified cfDNA using NonoDrop equipment. Notably, we found significant associations between cfDNA concentration and some clinical features such as stage, recurrence and metastatic potential. Although we found significant difference in the cfDNA concentration as measured by the mentioned commercially available kits, associations remained significant even when the reported concentrations were low (with MN method). This finding shows the validity of our obtained results. Moreover, cfDNA concentrations were not associated with patients’ age, sex or smoking history which shows the suitability of this source of biomarker for identification of disease status independent of these features. Although the cfDNA levels were associated with recurrence potential, after partial correction for patients’ gender, cfDNA concentrations were not correlated with recurrence time after initial diagnosis which might be explained by the relative low number of samples. Notably, cfDNA concentrations were not only predictive of metastatic potential, but also they could predict the number of involved organs which is potentially indicative of poor outcome. Based on the AUC values, diagnostic power of cfDNA concentration for prediction metastatic potential was higher than its ability to predict recurrence. Such diagnostic power was also independent of isolation method.

In brief, our study highlights the suitability of liquid biopsy as a non-invasive method for prediction of lung cancer prognosis in Iranian patients. As in the present study, we only assessed baseline cfDNA concentrations, future studies are needed to assess cfDNA levels in certain intervals after administration of drugs to explore their relevance with response of patients to each therapeutic regimens. We also showed availability of these methods for clinical practice and could solve the technical problems that were related to the evaluation of the cfDNA concentrations in the blood circulation. The similar results obtained from two mentioned kits indicate the reproducibility of the data.

## Conclusion

Based on the results of previous studies indicating that the diagnostic accuracy of quantitative investigation of cfDNA is not inferior to conventional peripheral biomarkers for lung cancer screening [12], such approaches can be used for initial screening of cancer patients as well. However, studies in large sample sizes are needed to explore the differences in cfDNA concentrations between normal individuals, precancerous conditions and cancer patients.

## Abbreviations

cf: circulating cell-free
SD: standard deviation
ROC: receiver operating characteristic
AUC: area under curve
